# Rehabilitation of the hemiparetic gait by nociceptive withdrawal reflex-based functional electrical therapy: a randomized, single-blinded study

**DOI:** 10.1186/1743-0003-11-81

**Published:** 2014-05-07

**Authors:** Erika Geraldina Spaich, Niels Svaneborg, Helle Rovsing Møller Jørgensen, Ole Kæseler Andersen

**Affiliations:** 1 Center for Sensory-Motor Interaction (SMI), Aalborg University, Fredrik Bajers Vej 7-D3, DK-9220 Aalborg, Denmark; 2 Brønderslev Neurorehabilitation Center, Vendsyssel Hospital, Nørregade 77, 9700 Brønderslev, Denmark

**Keywords:** Stroke, Gait rehabilitation, Nociceptive withdrawal reflex, Hemiparetic gait, Human locomotion, Reflex modulation

## Abstract

**Background:**

Gait deficits are very common after stroke and improved therapeutic interventions are needed. The objective of this study was therefore to investigate the therapeutic use of the nociceptive withdrawal reflex to support gait training in the subacute post-stroke phase.

**Methods:**

Individuals were randomly allocated to a treatment group that received physiotherapy-based gait training supported by withdrawal reflex stimulation and a control group that received physiotherapy-based gait training alone. Electrical stimuli delivered to the arch of the foot elicited the withdrawal reflex at heel-off with the purpose of facilitating the initiation and execution of the swing phase. Gait was assessed before and immediately after finishing treatment, and one month and six months after finishing treatment. Assessments included the Functional Ambulation Category (FAC) test, the preferred and maximum gait velocities, the duration of the stance phase in the hemiparetic side, the duration of the gait cycle, and the stance time symmetry ratio.

**Results:**

The treatment group showed an improved post treatment preferred walking velocity (p < 0.001) and fast walking velocity (p < 0.001) compared to the control group. Furthermore, subjects in the treatment group with severe walking impairment at inclusion time showed the best improvement as assessed by a longer duration of the stance phase in the hemiparetic side (p < 0.002) and a shorter duration of the gait cycle (p < 0.002). The stance time symmetry ratio was significantly better for the treatment than the control group after finishing training (p < 0.02). No differences between groups were detected with the FAC test after finishing training (p = 0.09).

**Conclusion:**

Withdrawal reflex-based functional electrical therapy was useful in the rehabilitation of the hemiparetic gait of severely impaired patients.

## Introduction

Regaining and maintaining the ability to walk independently is an important aspect of stroke rehabilitation and a primary goal for patients [1,2]. It concerns a large part of the stroke population, since approximately 50% of the patients have initially no walking function and 12% need assistance to walk [3]. Sixty percent of the initially non-ambulatory patients will regain independent walking after three months of training in a rehabilitation unit as compared to only 39% of those patients treated in an acute unit (see [4] for a review), which emphasizes the importance of the rehabilitation effort and course. The gait deficits post stroke include a range of spatio-temporal and kinematic deviations from normal gait, such as reduced speed and longer stance phase, reduced hip, knee, and ankle flexion during swing, and reduced knee extension during early-stance in the most affected side [5]. Gait-oriented training is commonly used after stroke and it has been shown to improve gait speed and walking distance [6]. Training typically consists of physiotherapy including the Bobath Concept, lower extremity muscle strengthening, overground walking, cycling, treadmill walking, and balance and cardiorespiratory training [7-14]. For a review on gait rehabilitation after stroke see [15].

Combining intensive voluntary exercising with synchronized functional electrical stimulation of the involved paretic muscles (functional electrical therapy, FET) has been of benefit for the recovery of upper-limb functions in the acute post-stroke phase [16] and to improve gait performance [17-19]. Additionally to the immediate effect of activating the appropriate muscles, the electrical stimulation ensures sensory inputs to the spinal and supraspinal centers by supporting the successful completion of the intended movements.

Stimulating relevant paretic muscles during gait therapy involves the activation of various muscle groups with the consequent tasks of positioning the stimulation electrodes, adjusting the stimulation intensities, and synchronizing the stimulation with the paretic gait pattern. Alternatively, ankle dorsiflexion, and in particular hip and knee flexion can be achieved by eliciting the withdrawal reflex [20-22]. Electrical stimulation of the sole of the foot has been shown to be effective to elicit the nociceptive withdrawal reflex (NWR) in the lower limb of healthy and hemiparetic individuals; the muscle and kinematic NWR responses were gait phase, stimulation site, frequency, and intensity dependent [23-26]. The characteristic kinematic response in hemiparetic individuals included dorsiflexion of the ankle joint and flexion of the knee and hip joints [25]. It has therefore been suggested that electric stimulation of the sole of the foot could be used to initiate and facilitate the swing phase of the hemiparetic gait [27]. The use of both an open-loop, single channel stimulator and a closed-loop system that selects the optimal stimulation parameters resulted in a more functional gait of hemiparetic individuals during a single training session [28], indicating that this modality of stimulation might benefit gait training.

The aim of the present study was therefore to investigate the therapeutic use of nociceptive withdrawal reflexes elicited from the sole of the foot to support the initiation and production of the swing phase and thereby gait training in the subacute post-stroke phase. The hypothesis was that combining intensive gait training with synchronized stimulation of the sole of the foot of the paretic limb would result in a lasting improvement of gait function after ending the treatment.

## Methods

### Subjects

The subjects that participated in this single-blinded study were 30 individuals who suffered a cerebrovascular accident (36–83 years old, see details in Table 1) and fulfilled the following inclusion criteria: age above 18, first ever cerebrovascular accident or second one with a first stroke with all neurological deficits fully resolved, cerebrovascular accident at most nine weeks old, able to walk a maximum of 10 meters without help from a therapist and eventually supporting on a bench or handrail if needed, ability to understand and follow instructions, able to tolerate electrical stimulation, no pacemaker, no heart or lung disease, no local infection in stimulation area, and no other neurologic or orthopedic problems affecting gait. Patients were recruited from a Neurorehabilitation center where they were admitted in the early subacute phase.

**Table 1:** Demographics of the subjects

**Patient**	**Age**	**Time since stroke (days)**	**Kind and location of CVA**	**Affected body side**	**Group**	**FAC-test score at inclusion**	**Mean training time [minutes]**	**Mean stimulation intensity [mA]**
1	60	28	Ischemic infarct – basal ganglia	left	NWR-FET	1	18.6	47.3
2	77	17	Ischemic infarct – corona radiata	right	Control	1	18.7	
3	76	32	Bilateral small ischemic infarcts in cerebrum - Ischemic infarct pons	left	NWR-FET	0	17.0	24.1
4	45	16	Ischemic infarct – area of middle cerebral artery	right	NWR-FET	0	18.3	18.6
5	50	31	Hemorrhage – intracerebral	right	Control	2	16.7	
6	62	34	Ischemic infarct – lentiform nucleus and lateral to the left lateral ventricle	right	Control	1	18.7	
7	70	62	Ischemic infarct – parieto-occipital lobe	left	NWR-FET	0	15.6	27.5
8	70	15	Ischemic infarct – lentiform nucleus and lateral to the left lateral ventricle	right	Control	2	18.1	
9	76	21	Ischemic infarct – area of middle cerebral artery	left	NWR-FET	2	17.5	9.3
10	71	13	Ischemic infarct – lentiform nucleus	left	Control	1	17.1	
11	77	19	Ischemic infarct – parietal lobe	right	NWR-FET	2	17.0	10.2
12	56	17	Ischemic infarct – corona radiata, temporal lobe and basal ganglia	left	Control	1	17.8	
13	77	33	Hemorrhage – intracerebral	left	NWR-FET	1	17.9	16.6
14	82	18	Ischemic infarct – area of middle cerebral artery	left	NWR-FET	1	16.2	12.4
15	62	14	Hemorrhage – basal ganglia	left	NWR-FET	1	17.6	13.1
16	83	23	Ischemic infarct – semioval center	right	Control	2	16.2	
17	68	29	Ischemic infarct – internal capsule	left	Control	1	16.5	
18	60	44	Hemorrhage – intracerebral	right	NWR-FET	0	16.8	33.3
19	75	62	Idiopathic CVA	right	Control	0	16.4	
20	39	62	Hemorrhage – intracerebral	right	NWR-FET	0	16.1	26.3
21	75	25	Ischemic infarct – external capsule	right	NWR-FET	2	16.6	22.9
22	57	40	Hemorrhage – thalamus	right	NWR-FET	2	15.9	14.6
23	75	23	Ischemic infarct – area of middle cerebral artery	right	NWR-FET	1	16.2	21.7
24	51	56	Ischemic infarct – area of middle cerebral artery	right	Control	0	15.5	
25	78	44	Ischemic infarct – insula, lentiform nucleus and caudal nucleus	right	Control	0	16.1	
26	58	36	Hemorrhage – intracerebral	left	Control	2	16.6	
27	79	20	Hemorrhage – external capsule	right	NWR-FET	2	16.8	20.0
28	66	16	Bilateral ischemic infarcts – cerebellum and brain stem	left	Control	0	17.9	
29	36	19	Ischemic infarct – area of middle cerebral artery	left	Control	0	17.3	
30	80	39	Ischemic infarct – pre- and post-central gyrus (left) parietal lobe (right)	right	Control	2	16.2	

The patients are numbered chronologically according to the date of inclusion in the study. The stimulation intensity corresponds to the average across 20 training sessions for the NWR-FET group. The mean training time corresponds to the average amount of minutes effectively used for training, excluding pauses, during the 20 training sessions. CVA: cerebrovascular accident.

The subjects were divided into two groups: a treatment group that received intensive physiotherapy-based gait training combined with activation of the NWR by electrical stimulation of the arch of the foot (NWR-FET group), and a control group that received intensive physiotherapy-based gait training alone. Concurrently with the study intervention, patients in both groups received physiotherapy five days a week, 40 minutes per day. Physiotherapy was based on a mixed approach and encompassed task specific repetitive training, gait training, the Bobath Concept, lower extremity muscle strengthening, and balance and cardiorespiratory training.

Computer-controlled randomization was performed at inclusion time in order to allocate the subjects into the two groups until two thirds of the total number of subjects was included. The remaining third was assigned to one of the two groups by computer software after the ambulation ability was assessed by the Functional Ambulation Category (FAC) test [29], in order to ensure balanced mobility at inclusion time. When performing the FAC test, patients are scored in a 6 point scale where, briefly, 0 corresponds to nonfunctional ambulation, 1 indicates need for continuous help to support body weight and balance, 2 indicates need for help to assist balance or coordination, 3 indicates ability to ambulate but need for supervision, 4 indicates ability to ambulate independently on level surfaces, and 5 corresponds to independent walkers on all surfaces [29].

The protocol of the study was approved by the local ethics committee (approval number VN-2004/65) and was in accordance with The Declaration of Helsinki. All volunteers provided written informed consent before participating in this study.

### Training protocol

Subjects in both groups received intensive gait training performed by physiotherapists, who determined the needs and training routines in an individualized manner. Training was considered intensive since it consisted of 20 daily sessions, with a maximum of two days without training between sessions. Furthermore, subjects trained during 30 minutes per day, with at least 15 minutes of walking, allowing for resting periods.

### NWR-FET

Individuals in the NWR-FET group received intensive physiotherapy-based gait training combined with activation of the nociceptive withdrawal reflex by electrical stimulation of the arch of the foot. The purpose of the stimulation was to elicit an unloading response of the affected limb and thereby support the initiation and production of the swing phase. To deliver the stimulation, a self-adhesive electrode (2.63 cm^2^ surface area, Ag-AgCl, Medicotest, Oelstykke, Denmark) was placed on the arch of the foot and a large common anode (7 × 10 cm electrode, Pals, Axelgaard Ltd., Fallbrook, California) was placed on the dorsum of the foot. The stimulation train consisted of five 1 ms-wide pulses delivered at 200 Hz, repeated 4 times at 15 Hz, triggered manually at heel-off. For this, a physiotherapist observed the patient attempt to lift-off and pressed a button that triggered the stimulation. The stimulation intensity was adjusted in steps of 1 mA by a physiotherapist that observed the overall evoked kinematic response and decided whether the stimulation intensity should be increased or decreased to evoke a stronger/weaker kinematic response that would best support the production of the swing phase.

### Evaluation

Evaluations were performed independently and blinded to therapy type at inclusion, immediately after completion of the 20 training sessions, and one and six months after completion of training.

During the evaluation sessions, the subjects were instrumented bilaterally with switches based on a force sensitive resistor (LuSense, PS3, Standard 174). The switches were placed under the heel and the medial forefoot, and used to record contact times while the subjects walked at their preferred velocity between one and four times along a 9 m long line. The recordings were sampled at 1 kHz, displayed on a computer screen, and stored for later analysis.

The ambulation ability was assessed by the FAC test. The preferred and maximum gait velocities were measured when walking along the 9 m long line. Measurements were performed between one and four times for each condition, averaged and stored. In both cases, the number of repetitions performed (1–4) depended solely on the physical condition of the subject.

Subjects walked barefoot and without walking aids such as ankle-foot orthosis or canes during the evaluation sessions. However, those subjects who needed the help of one or two therapists to walk were allowed to use it, with the constraint that the physiotherapist(s) should provide the minimum help necessary to allow walking and should not provide verbal guidance while performing the evaluations.

### Data analysis

The data analysis was performed off-line, independently, and blinded to therapy type. For visualization and processing of the footswitch data, ad-hoc software developed in Matlab (Matlab 7.14.0.739, The MathWorks Inc., Natick, MA, USA, 2012) was used. The data consisted of On-Off signals (contact/no-contact) that were visually inspected to identify and eliminate the sections lacking cyclic signals at the beginning and end of the recordings, indicating periods when the patients prepared to start walking and stopped walking after concluding the 9 m test. Afterwards, the data from the four switches were combined to generate the stance, swing, and single support phases. The duration of the stance phase and the single support phase of both limbs, and the gait cycle were measured. The duration of the stance and the single support phases were expressed as a percentage of the duration of the gait cycle. Outliers that fell outside the 5^th^ and 95^th^ percentiles were eliminated. For each subject, measurements corresponding to the duration of the stance phase in the ipsilateral, hemiparetic side and the gait cycle from the evaluation sessions performed immediately after, and one and six months after completion of training were normalized to the mean values at inclusion time in order to eliminate mismatches at inclusion time.

The amplitude of gait symmetry was assessed by means of a Symmetry Ratio calculated as the ratio between the duration of the stance time in the paretic limb and the stance time in the contralateral limb, provided that the larger of the two values is used in the numerator; gait with a Symmetry Ratio above the normative cut point of 1.05 is considered asymmetric [30].

### Statistical analysis

A mixed linear model analysis was performed for the preferred and maximum walking velocities, and the Symmetry Ratio with the following variables: group (NWR-FET and Control) and post training evaluation session (immediately after training, one month, and six months after training), and subject as a random factor. A similar analysis was performed for the duration of the stance phase in the hemiparetic side and the gait cycle but stratified according to the FAC-test score at inclusion time. T-test was used to compare the groups at inclusion time. Post-hoc comparisons were performed with the Bonferroni test. A generalized mixed linear model analysis was performed for the FAC-test score with the following variables: group (NWR-FET and Control) and post training evaluation session (immediately after training, one month, and six months after training), and subject as a random factor.

Furthermore, the Mann–Whitney Sum Rank test was used to test differences in age (data non-normally distributed) among groups at inclusion time. T-test was used to compare the training time among groups. To investigate changes of the stimulation intensity along the training period, a 1-way repeated measures ANOVA was used with week of training as variable.

Results are presented as mean values and standard error of the mean (SEM). P < 0.05 was considered statistically significant.

## Results

One subject (#20) dropped out of the study after the second evaluation session due to reasons unrelated to this project and one subject (#30) did not wish to participate in the last evaluation session. The data collected from these subjects were included in the analysis. At inclusion time, there was no statistically significant difference in the age of the subjects between groups (P = 0.7).

The mean training time for subjects in both groups and the mean stimulation intensity for the NWR-FET group are presented in Table 1. The mean training time across the 20 sessions was 16.9 minutes (range 15.6-18.6) for the NWR-FET group and 17.1 minutes (range 15.5-18.7) for the control group (P = 0.7). The mean stimulation intensities across subjects in the NWR-FET group for each of the four weeks of training were 22.1 ± 2.2 mA, 21.3 ± 2.6 mA, 20.6 ± 3.0 mA, and 20.6 ± 2.8 mA. No statistically significant change in the stimulation intensity during the training period was detected (ANOVA, P = 0.6). In the NWR-FET group, no cases of walking instability following the stimulation or desire to stop due to the short-lasting unpleasant stimulation were observed.

### General walking quality

At the first evaluation session, the subjects scored 0, 1, or 2 in the FAC-test. The three scores were equally distributed among subjects in both groups (NWR-FET and control). The test scores at the different evaluation sessions are presented in Table 2. The post training FAC-test score was influenced by the evaluation session in which it was assessed (Generalized mixed model analysis, main effect, p < 0.03) and there was no group effect (p = 0.09).

**Table 2:** Functional Ambulation Category test scores at the different evaluation sessions

	**Before training**	**Immediately after training**	**One month after training**	**Six months after training**
NWR-FET	1 [0; 2]	2 [2; 3]	4 [2; 5]	4.5 [2; 5]
Control	1 [0; 2]	2 [1; 2]	2 [2; 3]	4 [2; 5]

Data are presented as median and 25th and 75th percentiles in brackets.

**Figure 1:**
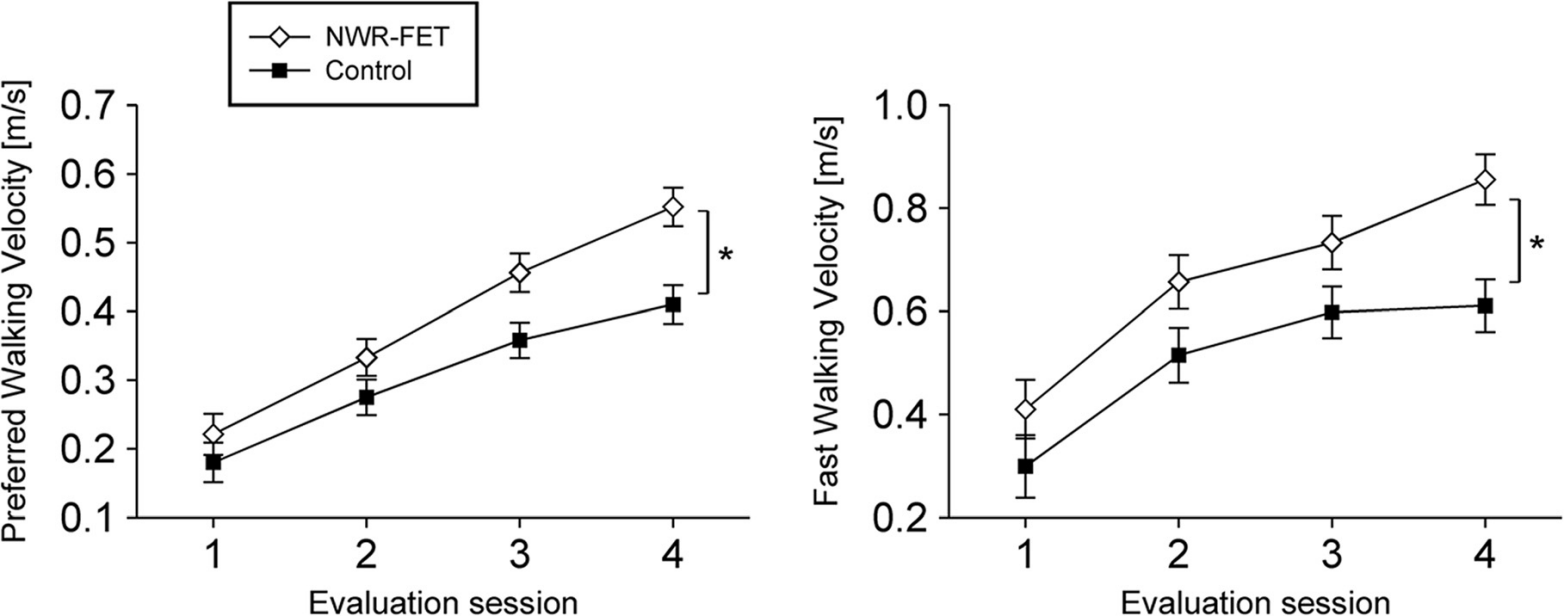
**Walking velocity.** Preferred and fast walking velocities at inclusion (evaluation 1), immediately after completion of training (evaluation 2), 1 month (evaluation 3), and 6 months (evaluation 4) after completion of training. Asterisk indicates a statistically significant difference between groups after finishing treatment (p < 0.001).

The preferred walking velocity was not statistically different between groups at inclusion time (p = 0.051) with a mean difference of 0.04 m/s. After training, the preferred walking velocity depended on the group that was assessed, with subjects in the NWR-FET group presenting a significantly faster preferred walking velocity (Mixed model analysis, main effect, p < 0.001) (Figure 1). The preferred walking velocity depended also on the evaluation session in which it was measured (Mixed model analysis, main effect, p < 0.001). Subjects improved their preferred walking velocity at each evaluation session post training (Bonferroni, p < 0.001).

The fast walking velocity was not statistically different between groups at inclusion time (p = 0.052) with a mean difference of 0.11 m/s. After training, it depended on the group that was assessed, with subjects in the NWR-FET group presenting a significantly faster maximum walking velocity (Mixed model analysis, main effect, p < 0.001) (Figure 1). The fast walking velocity depended also on the evaluation session in which it was measured (Mixed model analysis, main effect, p < 0.03). Subjects improved their fast walking velocity at each evaluation session post training (Bonferroni, p < 0.002).

### Gait parameters

For subjects in the FAC = 1 group at inclusion time, the post treatment duration of the stance phase in the hemiparetic side depended on the group to which the subjects belonged (NWR-FET/control) (Mixed model analysis, main effect, p < 0.001) with subjects in the NWR-FET group presenting a longer stance phase duration. Furthermore, for this stratum, the post treatment duration of the stance phase depended also on the evaluation session (Mixed model analysis, main effect, p < 0.001). Subjects shortened their stance phase duration from the evaluation immediately after finishing treatment to one month after (Bonferroni, p < 0.001).

**Figure 2:**
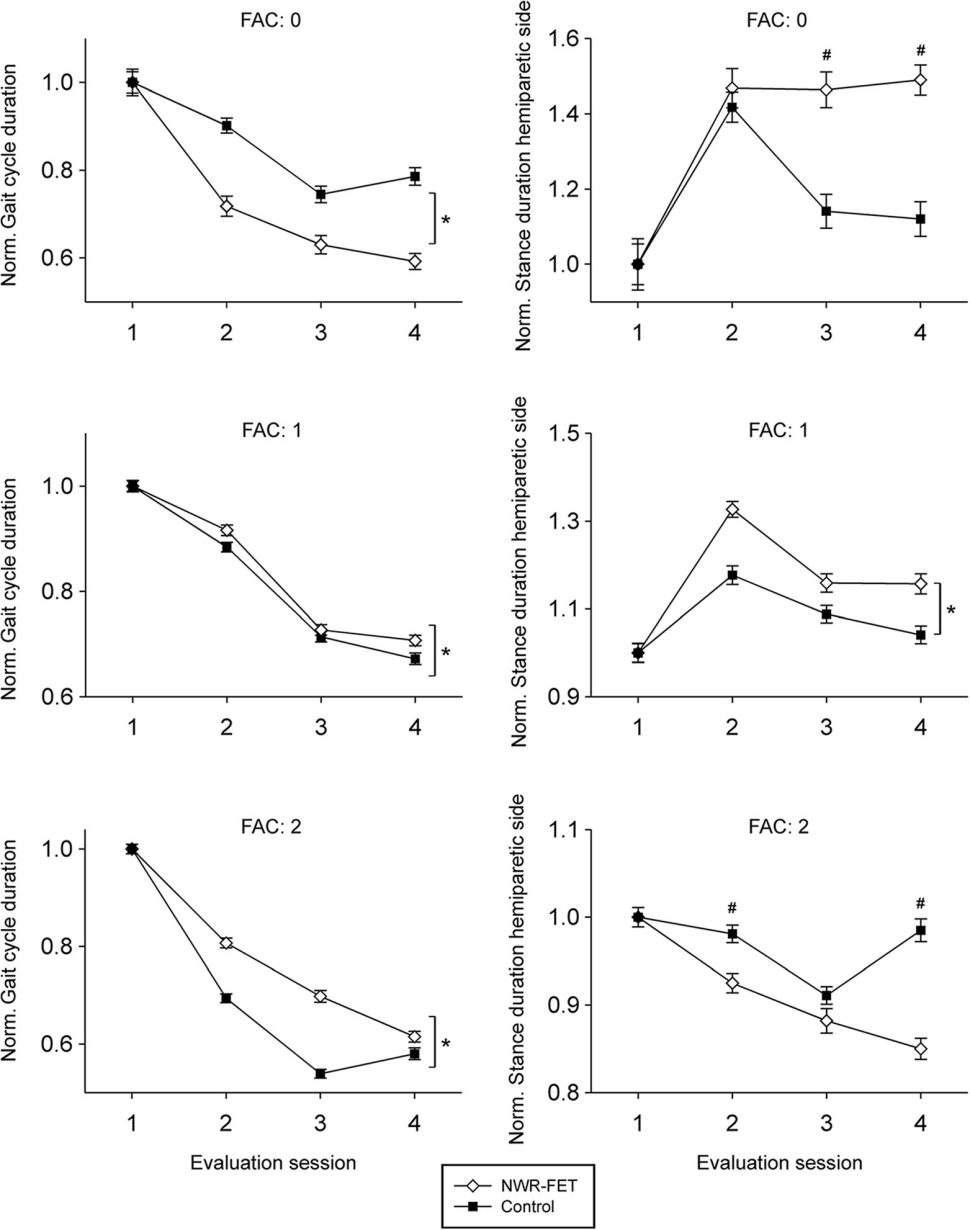
**Gait cycle and Stance phase.** Duration of the Gait cycle and of the Stance phase in the hemiparetic side stratified according to the FAC-test score at inclusion time (n = 5 for the NWR-FET and control groups for each FAC group). Values are normalized per subject to the mean at inclusion time to correct for differences at inclusion and shown at inclusion (evaluation 1), immediately after completion of training (evaluation 2), 1 month (evaluation 3), and 6 months (evaluation 4) after completion of training. Mean values at inclusion time are shown in Table 3. Asterisk indicates a statistically significant difference between groups after finishing treatment (p < 0.002). Square indicates a statistically significant interaction between group and evaluation session (p < 0.001).

For subjects in the FAC = 0 and FAC = 2 groups at inclusion time, the post treatment duration of the stance phase in the hemiparetic side depended on the group to which the subjects belonged (NWR-FET/control) and the evaluation session (Mixed model analysis, interaction, p < 0.002). Significant interactions (Bonferroni, p < 0.05) are shown in Figure 2 while the mean values at inclusion time are presented in Table 3.

**Table 3:** Duration of gait cycle and stance phase in the hemiparetic side at inclusion time

	**FAC: 0**	**FAC: 1**	**FAC: 2**
Gait cycle [s]			
NWR-FET	2.59 ± 0.08*	1.95 ± 0.03*	1.81 ± 0.03*
Control	3.25 ± 0.10	2.32 ± 0.03	2.51 ± 0.03
Stance phase (hemiparetic side) [% of gait cycle]			
NWR-FET	0.56 ± 0.01	0.61 ± 0.01	0.69 ± 0.01*
Control	0.51 ± 0.01	0.58 ± 0.01	0.60 ± 0.01

Data are presented as average across subjects ± SEM and stratified by FAC score at inclusion time. Asterisk indicates a statistically significant difference between groups (NWR-FET and Control) (p < 0.001).

The post treatment duration of the gait cycle depended on the group to which the subjects belonged (NWR-FET/control) (Mixed model analysis, main effect, p < 0.002). Subjects in the NWR-FET group, who scored 0 in the FAC test at inclusion, presented shorter post training gait cycle durations than subjects in the control group (Figure 2). Subjects in the NWR-FET group, who scored 1 and 2 in the FAC test at inclusion, presented instead longer post training gait cycle durations than subjects in the control group (Figure 2). The post treatment duration of the gait cycle depended also on the evaluation session (Mixed model analysis, main effect, p < 0.001). Subjects in the FAC = 1 group, shortened their gait cycle at each post training evaluation session (Bonferroni, p < 0.001), while subjects in the FAC = 0 and FAC = 2 groups, shortened their gait cycle only from the evaluation immediately after finishing treatment to one month after (Bonferroni, p < 0.001).

**Figure 3:**
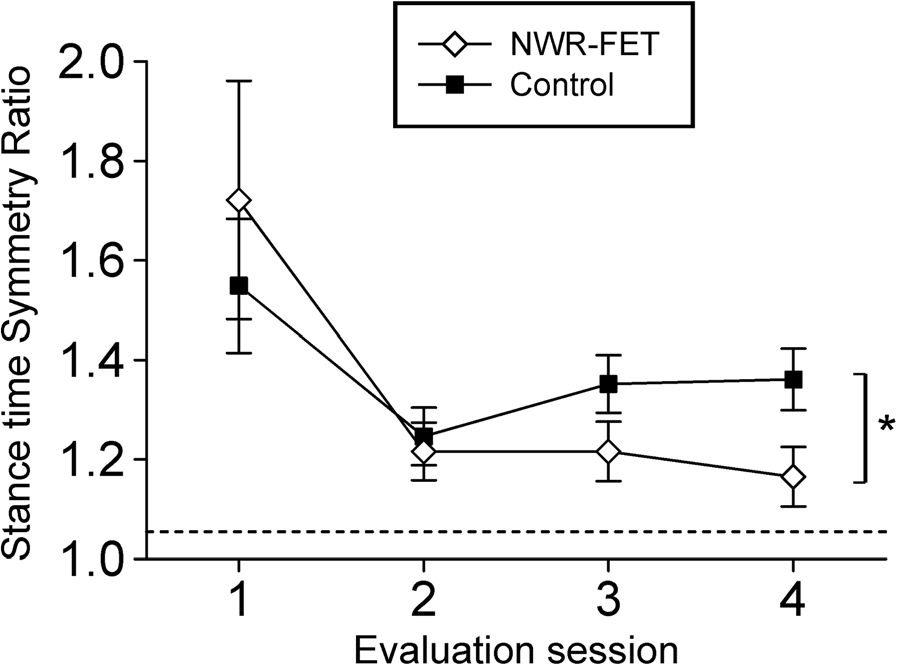
**Stance time Symmetry Ratio.** Symmetry Ratio at inclusion (evaluation 1), immediately after completion of training (evaluation 2), 1 month (evaluation 3), and 6 months (evaluation 4) after completion of training. The Symmetry Ratio was calculated as the ratio between the duration of the stance time in the paretic limb and the stance time in the contralateral limb, provided that the larger of the two values is used in the numerator. The stippled horizontal line represents the normative cut point of 1.05. Asterisk indicates a statistically significant difference between groups after finishing treatment (p < 0.02).

The stance time Symmetry Ratio was not statistically different between groups at inclusion time (p = 0.5) with a mean difference of 0.18. After training, the Symmetry Ratio depended on the group that was assessed, with subjects in the NWR-FET group presenting a significantly smaller Symmetry Ratio (Mixed model analysis, main effect, p < 0.02) that did however not fall below the normative cut point of 1.05 (Figure 3).

## Discussion

Intensive physiotherapy-based gait training combined with activation of the NWR to initiate and support the swing phase resulted in improved walking in subacute hemiparetic individuals, in particular those with severe walking impairment at inclusion time. This was shown by the faster preferred and fast walking velocities, the shortening of the gait cycle and lengthening of the stance phase on the affected side, and the improved Symmetry Ratio that tended towards values that indicate symmetric gait. The results suggest that withdrawal reflex-based therapy was useful in the rehabilitation of the hemiparetic gait.

### NWR-FET

In a study involving a single training session, it was shown that a more functional hemiparetic gait could be obtained by eliciting the lower limb NWR by electrical stimulation of the sole of the foot in order to obtain preset kinematic goals [28]. In the present study, the therapeutic effect of a NWR-FET system was studied and it was shown that although the system was very simple, consisting of an open-loop one channel stimulator, it succeeded to produce an effect that outlasted the training period. For the majority of the outcome measures, i.e. the preferred walking velocity, the fast walking velocity, the symmetry ratio, the duration of the gait cycle, and the duration of the stance phase for subjects in the FAC = 1 group, there was a post-training effect that depended on the group to which the subjects belonged, indicating an immediate but also a therapeutic effect of training with NWR-FET. Repeated functional exercising and the synchronized stimulations that facilitated the swing phase, creating a proprioceptive, sensory inflow to the supraspinal centers, might be responsible for this therapeutic effect. It is known that tactile stimulation results in an expanded representation of the stimulated areas in the somatosensory cortex of monkeys [31]. It is also known that in a monkey model of stroke, functional reorganization of the motor cortex is observed after a few weeks of training, suggesting that the adjacent, undamaged motor cortex might take over the function of the damaged cortex [32]. In humans, a long lasting reorganization of the motor cortex associated with improved motor performance following rehabilitation of the upper arm of stroke individuals has been reported [33]. Furthermore, results indicating that improved motor performance and increased motor cortex excitability followed skill training but not non-skill and passive training in humans [34] suggest that brain plasticity and perhaps also spinal cord plasticity (see [35] and [36] for reviews) might mediate the longer lasting changes in both the motor and somatosensory cortex representation of the lower limbs. This, in combination with the spinal pattern generation networks, would probably result in improved gait performance. It has been suggested that in animals, neuroplasticity is more effective during a time-limited window after stroke [37,38], which is consistent with results in humans indicating that better and faster recovery is obtained in the early phase after stroke [39]; therefore the rationale of including in this study individuals in the early sub-acute phase after stroke.

### Gait performance

Gait velocity has been shown to correlate to motor recovery [40] and although this measurement does not reflect the quality of the movements, it is affected by weakness in hip, knee, and ankle flexors [41,42]. In the present study, individuals who received NWR-FET showed a better improvement of their self-selected and fast walking velocities. However, results should be considered with caution, since most of the patients needed help from at least one physiotherapist to be able to perform the test, which is an important confounding factor due to the impossibility to standardize the amount of support provided by the therapist. Assessment of gait velocity of patients walking at their preferred velocity and using their own selection of walking aid or assistance has been reported earlier in the literature [43].

The minimal detectable change in gait speed of sub-acute stroke individuals who needed physical assistance from one person to walk has been reported to be 0.07 m/s [44], which suggests that the changes observed in the present study cannot only be explained by inherent data variability. On the other hand, the mean difference in the preferred walking velocity between groups at inclusion (0.04 m/s) could be attributed to the expected data variability [44]. The difference between groups became aproximately three times larger, increasing to 0.14 m/s at the last follow-up, indicating an effect of treatment. Although this difference seem small, it must be considered in light of the low walking velocity the subjects showed, specially at inclusion time. The preferred pre-training walking velocity was approximately 0.2 m/s revealing that the subjects included in this study had very limited ambulation abilities and could be classified as household walkers (<0.4 m/s) [45]. Individuals in the NWR-FES group changed from being classified as household walkers to becoming limited community walkers (0.4–0.8 m/s) one month after finishing training, showing an improvement in their walking ability. Individuals in the control group changed from household walkers to limited community walkers only six months after finishing training, revealing a slower pace of recovery. Although walking velocity is a good indicator of community ambulation, factors such as balance control and lower limb strength are also important to achieve independent walking [46].

Hemiparetic patients normally spend less time in single limb support and stance with their affected side [47]. The present results showed that gait training might change this, as the Symmetry Ratio tended towards the normative cut point indicating symmetric gait [30] after ending the treatment. Although none of the groups reached Symmetry Ratios lower than the normative cut point, the NWR-FET group obtained closer values, reflecting a positive effect of training. It was hypothesized that asymmetry in the stance time could be related to balance control issues that result in a shorter stance time in the hemiparetic side [30], which suggests that the gait training provided might have also been beneficial to improve balance.

Individuals in the NWR-FET group that were the poorest walkers at inclusion time obtained the largest benefit from treatment, reaching a final stride time approximately 40% shorter than at inclusion and a persistently longer stance time in the hemiparetic side of approximately 80% of the gait cycle. This result fits well with those showing that the relative duration of the gait phases is influenced by the gait speed and that at walking speeds lower than 0.7 m/s, as is the general case in the present study, the stance phase represents more than 80% of the gait cycle [48]. Individuals that had a better walking function at inclusion, FAC score of 1, and received NWR-FET also showed a longer duration of the stance phase in the hemiparetic side compared to those in the control group, indicating that a more normal gait pattern with better weight bearing in the affected side was achieved. However, those patients that were the best walkers at inclusion, FAC score of 3, did not seem to benefit as much from NWR-FET. They reduced the duration of the stance phase probably due to an earlier start of the swing phase caused by the electrical stimulus delivered at heel-off and shortened the gait cycle, though not as much as the individuals in the control group, reaching values comparable to those of healthy individuals [49]. It is therefore likely that the reflex stimulation might have perturbed the gait of these patients instead of supporting it. Finally, results from the last evaluation session, 6 months after ending the treatment, must be considered cautiously since patients had already been discharged from hospital which resulted in very heterogeneous courses of treatment, living conditions, and levels of activity across individuals.

### Experimental setup limitations

Activating the nociceptive withdrawal reflex requires using stimulation intensities that are perceived as unpleasant or painful [50]. This however was not a problem as indicated by informal patient verbal report and by the lack of dropouts. This suggests that patients might accept the associated discomfort if the treatment is limited in time (therapeutic use). Further aspects such as the appropriate treatment dose, automatic triggering of the stimulation and control of the stimulation intensity/location to avoid habituation of the reflex response [51] will require further investigations.

## Conclusion

In conclusion, this article presented a new therapeutic tool to rehabilitate gait consisting of intensive physiotherapy based gait training combined with activation of the nociceptive withdrawal reflex that improved the walking ability of hemiparetic patients resulting in a faster and more functional gait, especially for those individuals with very poor walking ability at inclusion time.

## Competing interests

EGS and OKA hold a patent (US8452410) and shares in a company (Nordic NeuroSTIM, ApS) relating to the contents of the manuscript.

## Authors’ contributions

EGS participated in the design and coordination of the study, acquisition, analysis, and interpretation of data, and drafted the manuscript. NS and HRMJ participated in the coordination of the study, recruitment of subjects, and revised critically the manuscript. OKA participated in the design of the study, acquisition of funding, interpretation of data, and revised critically the manuscript. All authors read and approved the final manuscript.

## References

[B1] Dobkin BH Clinical practice. Rehabilitation after stroke N Engl J Med 2005 352 1677 1684 10.1056/NEJMcp043511 15843670 PMC4106469

[B2] Bohannon RW Horton MG Wikholm JB Importance of four variables of walking to patients with stroke Int J Rehabil Res 1991 14 246 250 10.1097/00004356-199109000-00010 1938039

[B3] Jorgensen HS Nakayama H Raaschou HO Olsen TS Recovery of walking function in stroke patients: the Copenhagen Stroke Study Arch Phys Med Rehabil 1995 76 27 32 10.1016/S0003-9993(95)80038-7 7811170

[B4] Preston E Ada L Dean CM Stanton R Waddington G What is the probability of patients who are nonambulatory after stroke regaining independent walking? A systematic review Int J Stroke 2011 6 531 540 10.1111/j.1747-4949.2011.00668.x 22111798

[B5] Olney SJ Richards C Hemiparetic gait following stroke. Part I: Characteristics Gait & Posture 1996 4 136 148 10.1016/0966-6362(96)01063-6

[B6] van de Port IG Wood-Dauphinee S Lindeman E Kwakkel G Effects of exercise training programs on walking competency after stroke: a systematic review Am J Phys Med Rehabil 2007 86 935 951 10.1097/PHM.0b013e31802ee464 17303962

[B7] Richards CL Malouin F Wood-Dauphinee S Williams JI Bouchard JP Brunet D Task-specific physical therapy for optimization of gait recovery in acute stroke patients Arch Phys Med Rehabil 1993 74 612 620 10.1016/0003-9993(93)90159-8 8503751

[B8] Teixeira-Salmela LF Olney SJ Nadeau S Brouwer B Muscle strengthening and physical conditioning to reduce impairment and disability in chronic stroke survivors Arch Phys Med Rehabil 1999 80 1211 1218 10.1016/S0003-9993(99)90018-7 10527076

[B9] Ada L Dean CM Hall JM Bampton J Crompton S A treadmill and overground walking program improves walking in persons residing in the community after stroke: a placebo-controlled, randomized trial Arch Phys Med Rehabil 2003 84 1486 1491 10.1016/S0003-9993(03)00349-6 14586916

[B10] Dean CM Richards CL Malouin F Task-related circuit training improves performance of locomotor tasks in chronic stroke: a randomized, controlled pilot trial Arch Phys Med Rehabil 2000 81 409 417 10.1053/mr.2000.3839 10768528

[B11] Laufer Y Dickstein R Chefez Y Marcovitz E The effect of treadmill training on the ambulation of stroke survivors in the early stages of rehabilitation: a randomized study J Rehabil Res Dev 2001 38 69 78 11322472

[B12] Pohl M Werner C Holzgraefe M Kroczek G Mehrholz J Wingendorf I Hoölig G Koch R Hesse S Repetitive locomotor training and physiotherapy improve walking and basic activities of daily living after stroke: a single-blind, randomized multicentre trial (DEutsche GAngtrainerStudie, DEGAS) Clin Rehabil 2007 21 17 27 10.1177/0269215506071281 17213237

[B13] Eich HJ Mach H Werner C Hesse S Aerobic treadmill plus Bobath walking training improves walking in subacute stroke: a randomized controlled trial Clin Rehabil 2004 18 640 651 10.1191/0269215504cr779oa 15473116

[B14] Salbach NM Mayo NE Wood-Dauphinee S Hanley JA Richards CL Cote R A task-orientated intervention enhances walking distance and speed in the first year post stroke: a randomized controlled trial Clin Rehabil 2004 18 509 519 10.1191/0269215504cr763oa 15293485

[B15] Belda-Lois JM Mena-del HS Bermejo-Bosch I Moreno JC Pons JL Farina D Iosa M Molinari M Tamburella F Ramos A Caria A Solis-Escalante T Brunner C Rea M Rehabilitation of gait after stroke: a review towards a top-down approach J Neuroeng Rehabil 2011 8 66 84 10.1186/1743-0003-8-66 22165907 PMC3261106

[B16] Popovic MB Popovic DB Sinkjaer T Stefanovic A Schwirtlich L Clinical evaluation of Functional Electrical Therapy in acute hemiplegic subjects J Rehabil Res Dev 2003 40 443 453 10.1682/JRRD.2003.09.0443 15080229

[B17] Bogataj U Gros N Kljajic M Acimovic R Malezic M The rehabilitation of gait in patients with hemiplegia: a comparison between conventional therapy and multichannel functional electrical stimulation therapy Phys Ther 1995 75 490 502 7770495 10.1093/ptj/75.6.490

[B18] Daly JJ Roenigk K Holcomb J Rogers JM Butler K Gansen J McCabe J Fredrickson E Marsolais EB Ruff RL A randomized controlled trial of functional neuromuscular stimulation in chronic stroke subjects Stroke 2006 37 172 178 10.1161/01.STR.0000195129.95220.77 16322492

[B19] Stanic U Acimovic-Janezic R Gros N Trnkoczy A Bajd T Kljajic M Multichannel electrical stimulation for correction of hemiplegic gait. Methodology and preliminary results Scand J Rehabil Med 1978 10 75 92 307813

[B20] Klose KJ Jacobs PL Broton JG Guest RS Needham-Shropshire BM Lebwohl N Nash MS Green BA Evaluation of a training program for persons with SCI paraplegia using the Parastep 1 ambulation system: part 1. Ambulation performance and anthropometric measures Arch Phys Med Rehabil 1997 78 789 793 10.1016/S0003-9993(97)90188-X 9344294

[B21] Quintern J Bisle G Hartmann E Maier-Weiterschan C Bauduin G Flexion reflex in spastic hemiparesis: neurophysiological and therapeutic use3rd world congress in Neurological rehabilitation 2002 371 372

[B22] Quintern J Bisle G Hartmann E Maier-Weiterschan C Bauduin G Sofia GN Controlled clinical study of stimulation of flexor reflex afferents for gait rehabilitation in patients with hemiplegia From Basic Motor Control to Functional Recovery III 2003 Bulgaria: St. Kliment Ohridski University Press 240 246

[B23] Andersen OK Sonnenborg FA Arendt-Nielsen L Modular organization of human leg withdrawal reflexes elicited by electrical stimulation of the foot sole Muscle Nerve 1999 22 1520 1530 10.1002/(SICI)1097-4598(199911)22:11<1520::AID-MUS6>3.0.CO;2-V 10514229

[B24] Spaich EG Arendt-Nielsen L Andersen OK Modulation of lower limb withdrawal reflexes during gait: a topographical study J Neurophysiol 2004 91 258 266 12968008 10.1152/jn.00360.2003

[B25] Spaich EG Hinge HH Arendt-Nielsen L Andersen OK Modulation of the withdrawal reflex during hemiplegic gait: effect of stimulation site and gait phase Clin Neurophysiol 2006 117 2482 2495 10.1016/j.clinph.2006.07.139 16949341

[B26] Spaich EG Emborg J Collet T Arendt-Nielsen L Andersen OK Withdrawal reflex responses evoked by repetitive painful stimulation delivered on the sole of the foot during late stance: site, phase, and frequency modulation Exp Brain Res 2009 194 359 368 10.1007/s00221-009-1705-9 19189087

[B27] Lee KH Johnston R Electrically induced flexion reflex in gait training of hemiplegic patients: induction of the reflex Arch Phys Med Rehabil 1976 57 311 314 1084735

[B28] Emborg J Matjacic Z Bendtsen JD Spaich EG Cikajlo I Goljar N Andersen OK Design and test of a novel closed-loop system that exploits the nociceptive withdrawal reflex for swing-phase support of the hemiparetic gait IEEE Trans Biomed Eng 2011 58 960 970 21134806 10.1109/TBME.2010.2096507

[B29] Holden MK Gill KM Magliozzi MR Nathan J Piehl-Baker L Clinical gait assessment in the neurologically impaired Reliability and meaningfulness. Phys Ther 1984 64 35 40 10.1093/ptj/64.1.35 6691052

[B30] Patterson KK Gage WH Brooks D Black SE McIlroy WE Evaluation of gait symmetry after stroke: a comparison of current methods and recommendations for standardization Gait Posture 2010 31 241 246 10.1016/j.gaitpost.2009.10.014 19932621

[B31] Jenkins WM Merzenich MM Ochs MT Allard T Guic-Robles E Functional reorganization of primary somatosensory cortex in adult owl monkeys after behaviorally controlled tactile stimulation J Neurophysiol 1990 63 82 104 2299388 10.1152/jn.1990.63.1.82

[B32] Nudo RJ Remodeling of cortical motor representations after stroke: implications for recovery from brain damage Mol Psychiatry 1997 2 188 191 10.1038/sj.mp.4000188 9152980

[B33] Liepert J Bauder H Wolfgang HR Miltner WH Taub E Weiller C Treatment-induced cortical reorganization after stroke in humans Stroke 2000 31 1210 1216 10.1161/01.STR.31.6.1210 10835434

[B34] Perez MA Lungholt BK Nyborg K Nielsen JB Motor skill training induces changes in the excitability of the leg cortical area in healthy humans Exp Brain Res 2004 159 197 205 10.1007/s00221-004-1947-5 15549279

[B35] Duffau H Brain plasticity: From pathophysiological mechanisms to therapeutic applications J Clin Neurosci 2006 13 885 897 10.1016/j.jocn.2005.11.045 17049865

[B36] Wolpaw JR Spinal cord plasticity in acquisition and maintenance of motor skills Acta Physiologica 2007 189 155 169 10.1111/j.1748-1716.2006.01656.x 17250566

[B37] Murphy TH Corbett D Plasticity during stroke recovery: from synapse to behaviour Nature Reviews Neuroscience 2009 10 861 872 10.1038/nrn2735 19888284

[B38] Biernaskie J Chernenko G Corbett D Efficacy of rehabilitative experience declines with time after focal ischemic brain injury J Neurosci 2004 24 1245 1254 10.1523/JNEUROSCI.3834-03.2004 14762143 PMC6793570

[B39] Sabut SK Sikdar C Kumar R Mahadevappa M Improvement of gait & muscle strength with functional electrical stimulation in sub-acute & chronic stroke patients Conf Proc IEEE Eng Med Biol Soc 2011 2011 2085 2088 22254748 10.1109/IEMBS.2011.6090387

[B40] Brandstater ME de Bruin H Gowland C Clark BM Hemiplegic gait: analysis of temporal variables Arch Phys Med Rehabil 1983 64 583 587 6661021

[B41] Hsu AL Tang PF Jan MH Analysis of impairments influencing gait velocity and asymmetry of hemiplegic patients after mild to moderate stroke Arch Phys Med Rehabil 2003 84 1185 1193 10.1016/S0003-9993(03)00030-3 12917858

[B42] Lin PY Yang YR Cheng SJ Wang RY The relation between ankle impairments and gait velocity and symmetry in people with stroke Arch Phys Med Rehabil 2006 87 562 568 10.1016/j.apmr.2005.12.042 16571398

[B43] Collen FM Wade DT Bradshaw CM Mobility after stroke: reliability of measures of impairment and disability Int Disabil Stud 1990 12 6 9 10.3109/03790799009166594 2211468

[B44] Fulk GD Echternach JL Test-retest reliability and minimal detectable change of gait speed in individuals undergoing rehabilitation after stroke J Neurol Phys Ther 2008 32 8 13 10.1097/NPT0b013e31816593c0 18463550

[B45] Taylor-Piliae RE Latt LD Hepworth JT Coull BM Predictors of gait velocity among community-dwelling stroke survivors Gait Posture 2012 35 395 399 10.1016/j.gaitpost.2011.10.358 22119886 PMC4696768

[B46] Kollen B Kollen B van de Port I Lindeman E Twisk J Kwakkel G Predicting improvement in gait after stroke: a longitudinal prospective study Stroke 2005 36 2676 2680 10.1161/01.STR.0000190839.29234.50 16282540

[B47] von Schroeder HP Coutts RD Lyden PD Billings E Jr Nickel VL Gait parameters following stroke: a practical assessment J Rehabil Res Dev 1995 32 25 31 7760264

[B48] van Hedel HJ Tomatis L Muller R Modulation of leg muscle activity and gait kinematics by walking speed and bodyweight unloading Gait Posture 2006 24 35 45 10.1016/j.gaitpost.2005.06.015 16099161

[B49] Chen CL Chen HC Tang SF Wu CY Cheng PT Hong WH Gait performance with compensatory adaptations in stroke patients with different degrees of motor recovery Am J Phys Med Rehabil 2003 82 925 935 10.1097/01.PHM.0000098040.13355.B5 14627929

[B50] Andersen OK Spaich EG Madeleine P Arendt-Nielsen L Gradual enlargement of human withdrawal reflex receptive fields following repetitive painful stimulation Brain Res 2005 1042 194 204 10.1016/j.brainres.2005.02.039 15854591

[B51] Dimitrijevic MR Nathan PW Studies of spasticity in man. 5. dishabituation of the flexion reflex in spinal man Brain 1971 94 77 90 10.1093/brain/94.1.77 4251710

